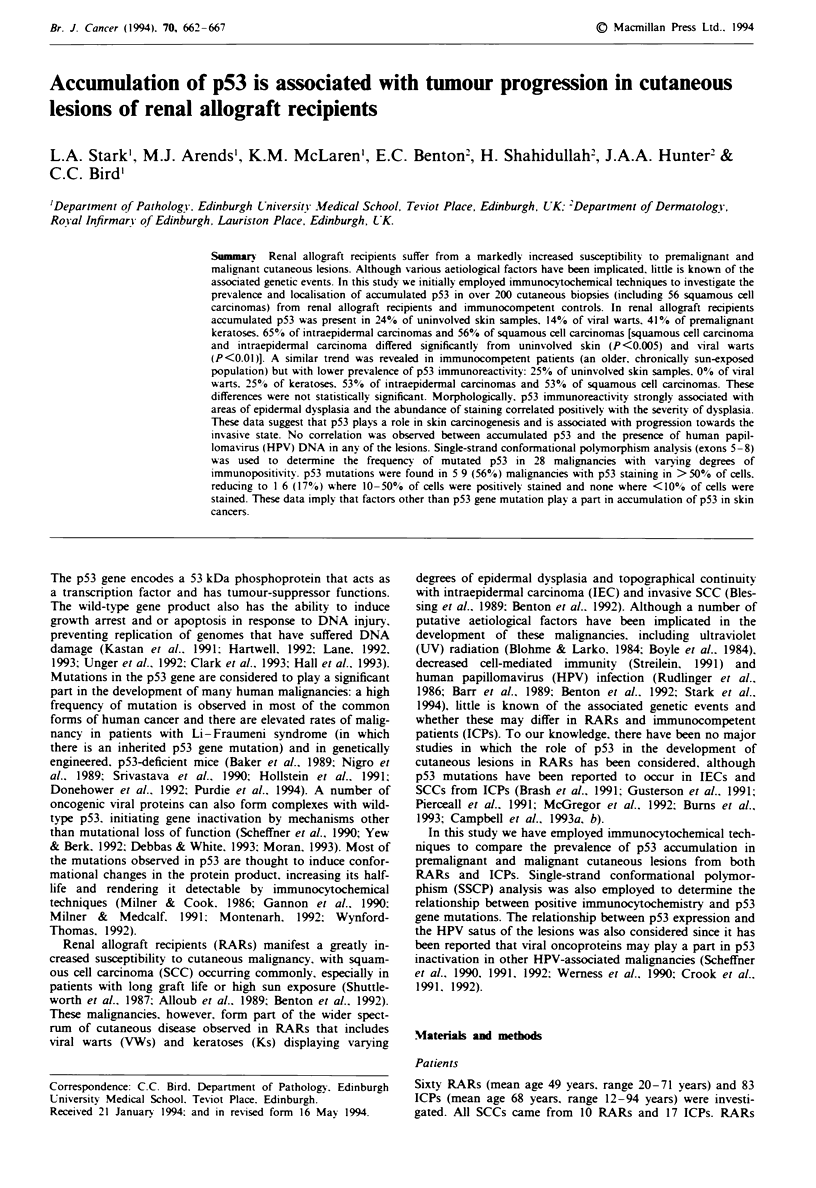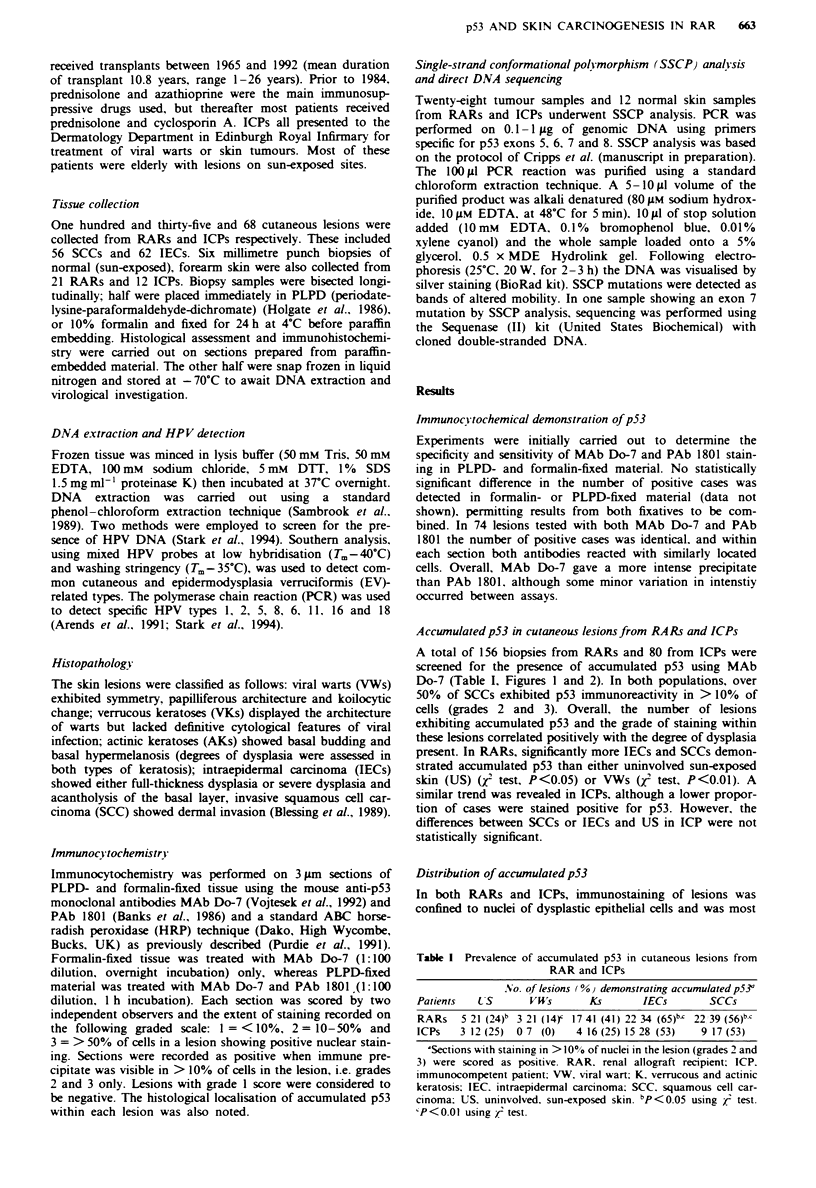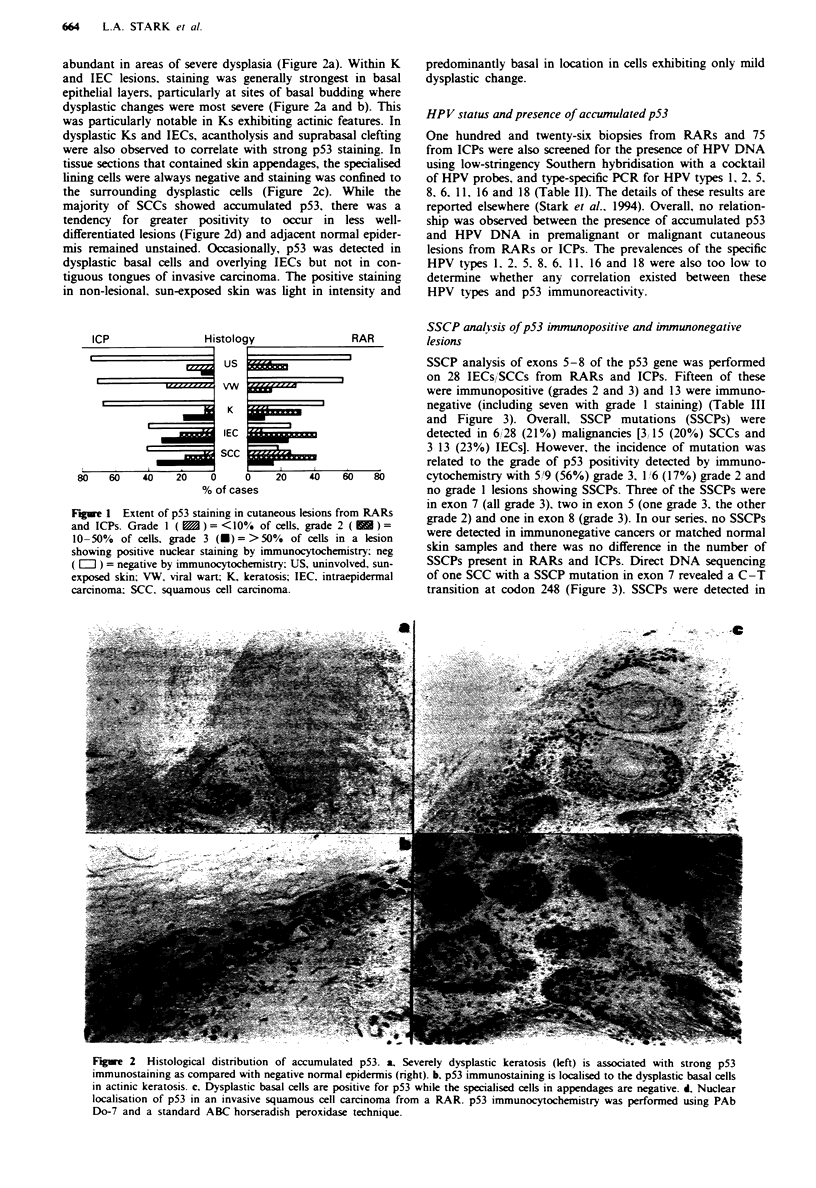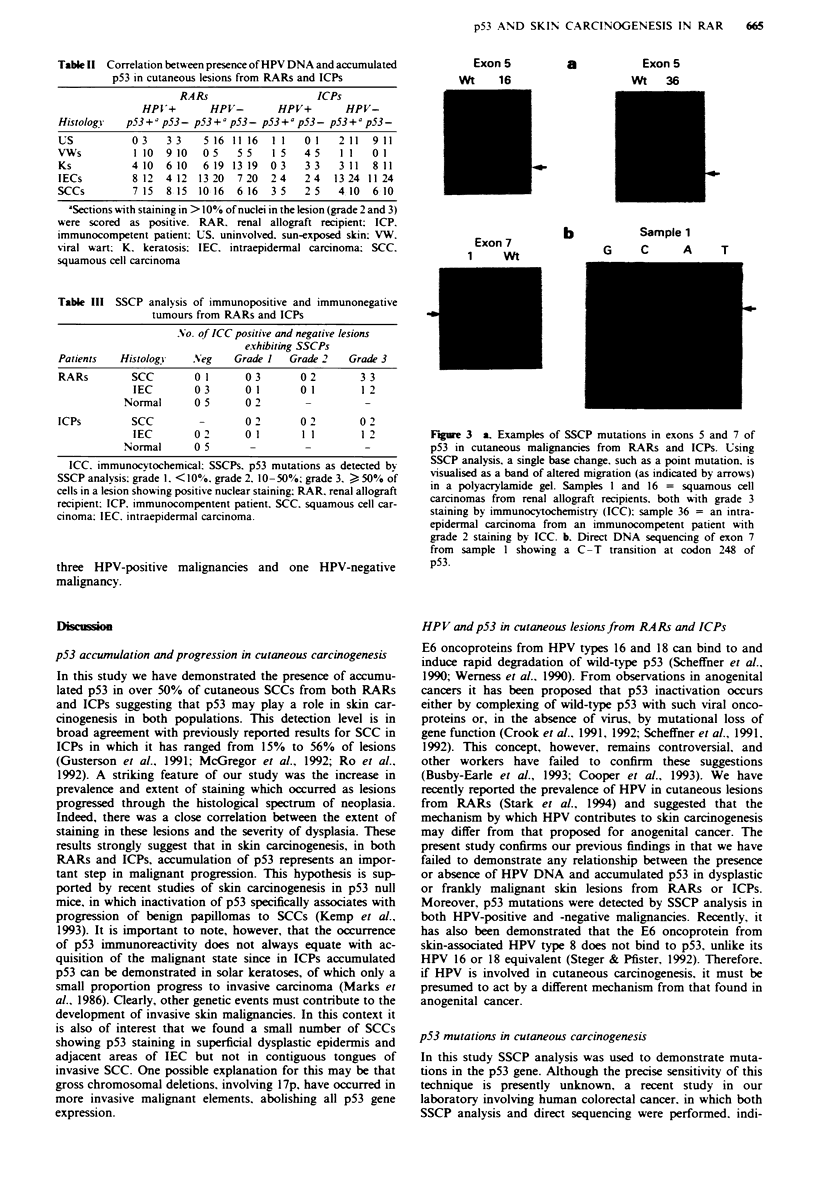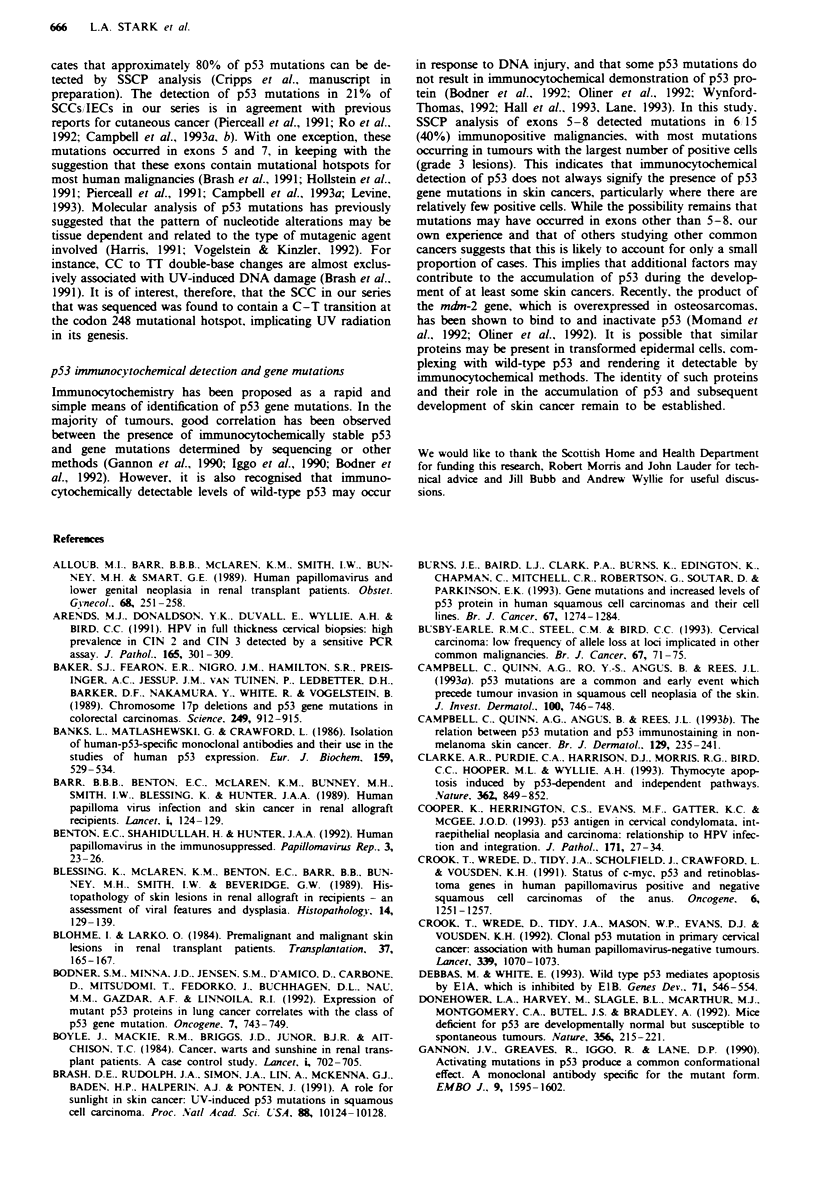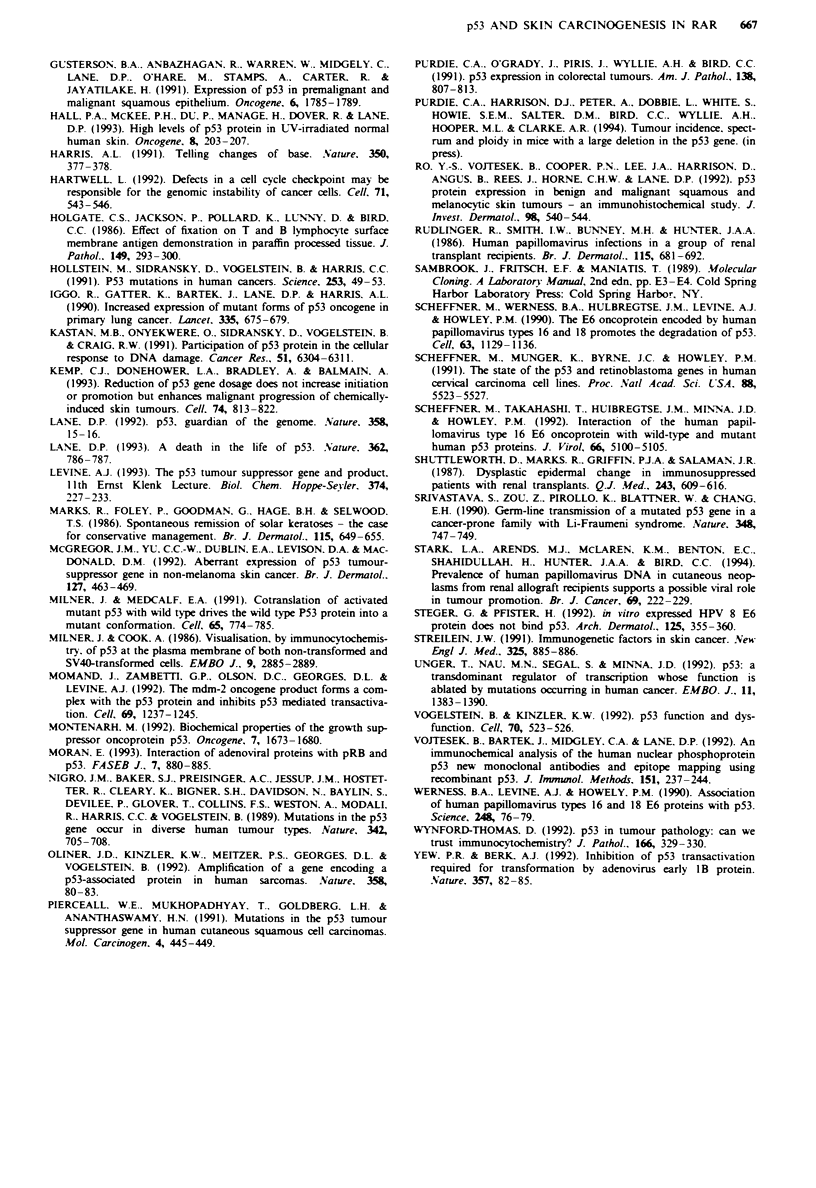# Accumulation of p53 is associated with tumour progression in cutaneous lesions of renal allograft recipients.

**DOI:** 10.1038/bjc.1994.367

**Published:** 1994-10

**Authors:** L. A. Stark, M. J. Arends, K. M. McLaren, E. C. Benton, H. Shahidullah, J. A. Hunter, C. C. Bird

**Affiliations:** Department of Pathology, Edinburgh University Medical School, UK.

## Abstract

**Images:**


Br. J. Cancer (1994), 70, 662-667

© Macmillan Press Ltd., 1994

### Accumulation of p53 is associated with tumour progression in cutaneous lesions of renal allograft recipients

L.A. Stark[1], M.J. Arends[1], K.M. McLaren[1], E.C. Benton[2], H. Shahidullah[2], J.A.A. Hunter[2] & C.C. Bird[1]

[1]*Department of Pathology, Edinburgh University Medical School, Teviot Place, Edinburgh, UK;* [2]*Department of Dermatology, Royal Infirmary of Edinburgh, Lauriston Place, Edinburgh, UK.*

**Summary** Renal allograft recipients suffer from a markedly increased susceptibility to premalignant and malignant cutaneous lesions. Although various aetiological factors have been implicated, little is known of the associated genetic events. In this study we initially employed immunocytochemical techniques to investigate the prevalence and localisation of accumulated p53 in over 200 cutaneous biopsies (including 56 squamous cell carcinomas) from renal allograft recipients and immunocompetent controls. In renal allograft recipients accumulated p53 was present in 24% of uninvolved skin samples, 14% of viral warts, 41% of premalignant keratoses, 65% of intraepidermal carcinomas and 56% of squamous cell carcinomas [squamous cell carcinoma and intraepidermal carcinoma differed significantly from uninvolved skin ($P<0.005$) and viral warts ($P<0.01$)]. A similar trend was revealed in immunocompetent patients (an older, chronically sun-exposed population) but with lower prevalence of p53 immunoreactivity: 25% of uninvolved skin samples, 0% of viral warts, 25% of keratoses, 53% of intraepidermal carcinomas and 53% of squamous cell carcinomas. These differences were not statistically significant. Morphologically, p53 immunoreactivity strongly associated with areas of epidermal dysplasia and the abundance of staining correlated positively with the severity of dysplasia. These data suggest that p53 plays a role in skin carcinogenesis and is associated with progression towards the invasive state. No correlation was observed between accumulated p53 and the presence of human papillomavirus (HPV) DNA in any of the lesions. Single-strand conformational polymorphism analysis (exons 5-8) was used to determine the frequency of mutated p53 in 28 malignancies with varying degrees of immunopositivity. p53 mutations were found in 5 9 (56%) malignancies with p53 staining in >50% of cells, reducing to 1 6 (17%) where 10-50% of cells were positively stained and none where <10% of cells were stained. These data imply that factors other than p53 gene mutation play a part in accumulation of p53 in skin cancers.

The p53 gene encodes a 53 kDa phosphoprotein that acts as a transcription factor and has tumour-suppressor functions. The wild-type gene product also has the ability to induce growth arrest and or apoptosis in response to DNA injury, preventing replication of genomes that have suffered DNA damage (Kastan *et al.*, 1991; Hartwell, 1992; Lane, 1992, 1993; Unger *et al.*, 1992; Clark *et al.*, 1993; Hall *et al.*, 1993). Mutations in the p53 gene are considered to play a significant part in the development of many human malignancies: a high frequency of mutation is observed in most of the common forms of human cancer and there are elevated rates of malignancy in patients with Li-Fraumeni syndrome (in which there is an inherited p53 gene mutation) and in genetically engineered, p53-deficient mice (Baker *et al.*, 1989; Nigro *et al.*, 1989; Srivastava *et al.*, 1990; Hollstein *et al.*, 1991; Donehower *et al.*, 1992; Purdie *et al.*, 1994). A number of oncogenic viral proteins can also form complexes with wild-type p53, initiating gene inactivation by mechanisms other than mutational loss of function (Scheffner *et al.*, 1990; Yew & Berk, 1992; Debbas & White, 1993; Moran, 1993). Most of the mutations observed in p53 are thought to induce conformational changes in the protein product, increasing its half-life and rendering it detectable by immunocytochemical techniques (Milner & Cook, 1986; Gannon *et al.*, 1990; Milner & Medcalf, 1991; Montenarh, 1992; Wynford-Thomas, 1992).

Renal allograft recipients (RARs) manifest a greatly increased susceptibility to cutaneous malignancy, with squamous cell carcinoma (SCC) occurring commonly, especially in patients with long graft life or high sun exposure (Shuttleworth *et al.*, 1987; Alloub *et al.*, 1989; Benton *et al.*, 1992). These malignancies, however, form part of the wider spectrum of cutaneous disease observed in RARs that includes viral warts (VWs) and keratoses (Ks) displaying varying degrees of epidermal dysplasia and topographical continuity with intraepidermal carcinoma (IEC) and invasive SCC (Blessing *et al.*, 1989; Benton *et al.*, 1992). Although a number of putative aetiological factors have been implicated in the development of these malignancies, including ultraviolet (UV) radiation (Blohme & Larko, 1984; Boyle *et al.*, 1984), decreased cell-mediated immunity (Streilein, 1991) and human papillomavirus (HPV) infection (Rudlinger *et al.*, 1986; Barr *et al.*, 1989; Benton *et al.*, 1992; Stark *et al.*, 1994), little is known of the associated genetic events and whether these may differ in RARs and immunocompetent patients (ICPs). To our knowledge, there have been no major studies in which the role of p53 in the development of cutaneous lesions in RARs has been considered, although p53 mutations have been reported to occur in IECs and SCCs from ICPs (Brash *et al.*, 1991; Gusterson *et al.*, 1991; Pierceall *et al.*, 1991; McGregor *et al.*, 1992; Burns *et al.*, 1993; Campbell *et al.*, 1993*a*, *b*).

In this study we have employed immunocytochemical techniques to compare the prevalence of p53 accumulation in premalignant and malignant cutaneous lesions from both RARs and ICPs. Single-strand conformational polymorphism (SSCP) analysis was also employed to determine the relationship between positive immunocytochemistry and p53 gene mutations. The relationship between p53 expression and the HPV satus of the lesions was also considered since it has been reported that viral oncoproteins may play a part in p53 inactivation in other HPV-associated malignancies (Scheffner *et al.*, 1990, 1991, 1992; Werness *et al.*, 1990; Crook *et al.*, 1991, 1992).

### Materials and methods

### *Patients*

Sixty RARs (mean age 49 years, range 20-71 years) and 83 ICPs (mean age 68 years, range 12-94 years) were investigated. All SCCs came from 10 RARs and 17 ICPs. RARs

Correspondence: C.C. Bird, Department of Pathology, Edinburgh University Medical School, Teviot Place, Edinburgh.
Received 21 January 1994; and in revised form 16 May 1994.

received transplants between 1965 and 1992 (mean duration of transplant 10.8 years, range 1–26 years). Prior to 1984, prednisolone and azathioprine were the main immunosuppressive drugs used, but thereafter most patients received prednisolone and cyclosporin A. ICPs all presented to the Dermatology Department in Edinburgh Royal Infirmary for treatment of viral warts or skin tumours. Most of these patients were elderly with lesions on sun-exposed sites.

*Tissue collection*

One hundred and thirty-five and 68 cutaneous lesions were collected from RARs and ICPs respectively. These included 56 SCCs and 62 IECs. Six millimetre punch biopsies of normal (sun-exposed), forearm skin were also collected from 21 RARs and 12 ICPs. Biopsy samples were bisected longitudinally; half were placed immediately in PLPD (periodate-lysine-paraformaldehyde-dichromate) (Holgate *et al.*, 1986), or 10% formalin and fixed for 24 h at 4°C before paraffin embedding. Histological assessment and immunohistochemistry were carried out on sections prepared from paraffin-embedded material. The other half were snap frozen in liquid nitrogen and stored at −70°C to await DNA extraction and virological investigation.

*DNA extraction and HPV detection*

Frozen tissue was minced in lysis buffer (50 mM Tris, 50 mM EDTA, 100 mM sodium chloride, 5 mM DTT, 1% SDS 1.5 mg ml$^{-1}$ proteinase K) then incubated at 37°C overnight. DNA extraction was carried out using a standard phenol–chloroform extraction technique (Sambrook *et al.*, 1989). Two methods were employed to screen for the presence of HPV DNA (Stark *et al.*, 1994). Southern analysis, using mixed HPV probes at low hybridisation ($T_m$ − 40°C) and washing stringency ($T_m$ − 35°C), was used to detect common cutaneous and epidermodysplasia verruciformis (EV)-related types. The polymerase chain reaction (PCR) was used to detect specific HPV types 1, 2, 5, 8, 6, 11, 16 and 18 (Arends *et al.*, 1991; Stark *et al.*, 1994).

*Histopathology*

The skin lesions were classified as follows: viral warts (VWs) exhibited symmetry, papilliferous architecture and koilocytic change; verrucous keratoses (VKs) displayed the architecture of warts but lacked definitive cytological features of viral infection; actinic keratoses (AKs) showed basal budding and basal hypermelanosis (degrees of dysplasia were assessed in both types of keratosis); intraepidermal carcinoma (IECs) showed either full-thickness dysplasia or severe dysplasia and acantholysis of the basal layer, invasive squamous cell carcinoma (SCC) showed dermal invasion (Blessing *et al.*, 1989).

*Immunocytochemistry*

Immunocytochemistry was performed on 3 μm sections of PLPD- and formalin-fixed tissue using the mouse anti-p53 monoclonal antibodies MAb Do-7 (Vojtesek *et al.*, 1992) and PAb 1801 (Banks *et al.*, 1986) and a standard ABC horseradish peroxidase (HRP) technique (Dako, High Wycombe, Bucks, UK) as previously described (Purdie *et al.*, 1991). Formalin-fixed tissue was treated with MAb Do-7 (1:100 dilution, overnight incubation) only, whereas PLPD-fixed material was treated with MAb Do-7 and PAb 1801 (1:100 dilution, 1 h incubation). Each section was scored by two independent observers and the extent of staining recorded on the following graded scale: 1 = <10%, 2 = 10–50% and 3 = >50% of cells in a lesion showing positive nuclear staining. Sections were recorded as positive when immune precipitate was visible in >10% of cells in the lesion, i.e. grades 2 and 3 only. Lesions with grade 1 score were considered to be negative. The histological localisation of accumulated p53 within each lesion was also noted.

*Single-strand conformational polymorphism (SSCP) analysis and direct DNA sequencing*

Twenty-eight tumour samples and 12 normal skin samples from RARs and ICPs underwent SSCP analysis. PCR was performed on 0.1–1 μg of genomic DNA using primers specific for p53 exons 5, 6, 7 and 8. SSCP analysis was based on the protocol of Cripps *et al.* (manuscript in preparation). The 100 μl PCR reaction was purified using a standard chloroform extraction technique. A 5–10 μl volume of the purified product was alkali denatured (80 μM sodium hydroxide, 10 μM EDTA, at 48°C for 5 min), 10 μl of stop solution added (10 mM EDTA, 0.1% bromophenol blue, 0.01% xylene cyanol) and the whole sample loaded onto a 5% glycerol, 0.5 × MDE Hydrolink gel. Following electrophoresis (25°C, 20 W, for 2–3 h) the DNA was visualised by silver staining (BioRad kit). SSCP mutations were detected as bands of altered mobility. In one sample showing an exon 7 mutation by SSCP analysis, sequencing was performed using the Sequenase (II) kit (United States Biochemical) with cloned double-stranded DNA.

### Results

*Immunocytochemical demonstration of p53*

Experiments were initially carried out to determine the specificity and sensitivity of MAb Do-7 and PAb 1801 staining in PLPD- and formalin-fixed material. No statistically significant difference in the number of positive cases was detected in formalin- or PLPD-fixed material (data not shown), permitting results from both fixatives to be combined. In 74 lesions tested with both MAb Do-7 and PAb 1801 the number of positive cases was identical, and within each section both antibodies reacted with similarly located cells. Overall, MAb Do-7 gave a more intense precipitate than PAb 1801, although some minor variation in intenstiy occurred between assays.

*Accumulated p53 in cutaneous lesions from RARs and ICPs*

A total of 156 biopsies from RARs and 80 from ICPs were screened for the presence of accumulated p53 using MAb Do-7 (Table I, Figures 1 and 2). In both populations, over 50% of SCCs exhibited p53 immunoreactivity in >10% of cells (grades 2 and 3). Overall, the number of lesions exhibiting accumulated p53 and the grade of staining within these lesions correlated positively with the degree of dysplasia present. In RARs, significantly more IECs and SCCs demonstrated accumulated p53 than either uninvolved sun-exposed skin (US) ($\chi^2$ test, $P<0.05$) or VWs ($\chi^2$ test, $P<0.01$). A similar trend was revealed in ICPs, although a lower proportion of cases were stained positive for p53. However, the differences between SCCs or IECs and US in ICP were not statistically significant.

*Distribution of accumulated p53*

In both RARs and ICPs, immunostaining of lesions was confined to nuclei of dysplastic epithelial cells and was most

**Table I** Prevalence of accumulated p53 in cutaneous lesions from RAR and ICPs

| | *No. of lesions (%) demonstrating accumulated p53*[a] | | | | |
|---|---|---|---|---|---|
| *Patients* | *US* | *VWs* | *Ks* | *IECs* | *SCCs* |
| RARs | 5/21 (24)[b] | 3/21 (14)[c] | 17/41 (41) | 22/34 (65)[b,c] | 22/39 (56)[b,c] |
| ICPs | 3/12 (25) | 0/7 (0) | 4/16 (25) | 15/28 (53) | 9/17 (53) |

[a]Sections with staining in >10% of nuclei in the lesion (grades 2 and 3) were scored as positive. RAR, renal allograft recipient; ICP, immunocompetent patient; VW, viral wart; K, verrucous and actinic keratosis; IEC, intraepidermal carcinoma; SCC, squamous cell carcinoma; US, uninvolved, sun-exposed skin. [b]$P<0.05$ using $\chi^2$ test. [c]$P<0.01$ using $\chi^2$ test.

abundant in areas of severe dysplasia (Figure 2a). Within K and IEC lesions, staining was generally strongest in basal epithelial layers, particularly at sites of basal budding where dysplastic changes were most severe (Figure 2a and b). This was particularly notable in Ks exhibiting actinic features. In dysplastic Ks and IECs, acantholysis and suprabasal clefting were also observed to correlate with strong p53 staining. In tissue sections that contained skin appendages, the specialised lining cells were always negative and staining was confined to the surrounding dysplastic cells (Figure 2c). While the majority of SCCs showed accumulated p53, there was a tendency for greater positivity to occur in less well-differentiated lesions (Figure 2d) and adjacent normal epidermis remained unstained. Occasionally, p53 was detected in dysplastic basal cells and overlying IECs but not in contiguous tongues of invasive carcinoma. The positive staining in non-lesional, sun-exposed skin was light in intensity and predominantly basal in location in cells exhibiting only mild dysplastic change.

**Figure 1** Extent of p53 staining in cutaneous lesions from RARs and ICPs. Grade 1 ( ▨ ) = <10% of cells, grade 2 ( ▩ ) = 10–50% of cells, grade 3 (■) = >50% of cells in a lesion showing positive nuclear staining by immunocytochemistry; neg ( □ ) = negative by immunocytochemistry; US, uninvolved, sun-exposed skin; VW, viral wart; K, keratosis; IEC, intraepidermal carcinoma; SCC, squamous cell carcinoma.

### *HPV status and presence of accumulated p53*

One hundred and twenty-six biopsies from RARs and 75 from ICPs were also screened for the presence of HPV DNA using low-stringency Southern hybridisation with a cocktail of HPV probes, and type-specific PCR for HPV types 1, 2, 5, 8, 6, 11, 16 and 18 (Table II). The details of these results are reported elsewhere (Stark *et al.*, 1994). Overall, no relationship was observed between the presence of accumulated p53 and HPV DNA in premalignant or malignant cutaneous lesions from RARs or ICPs. The prevalences of the specific HPV types 1, 2, 5, 8, 6, 11, 16 and 18 were also too low to determine whether any correlation existed between these HPV types and p53 immunoreactivity.

### *SSCP analysis of p53 immunopositive and immunonegative lesions*

SSCP analysis of exons 5–8 of the p53 gene was performed on 28 IECs/SCCs from RARs and ICPs. Fifteen of these were immunopositive (grades 2 and 3) and 13 were immunonegative (including seven with grade 1 staining) (Table III and Figure 3). Overall, SSCP mutations (SSCPs) were detected in 6/28 (21%) malignancies [3/15 (20%) SCCs and 3/13 (23%) IECs]. However, the incidence of mutation was related to the grade of p53 positivity detected by immunocytochemistry with 5/9 (56%) grade 3, 1/6 (17%) grade 2 and no grade 1 lesions showing SSCPs. Three of the SSCPs were in exon 7 (all grade 3), two in exon 5 (one grade 3, the other grade 2) and one in exon 8 (grade 3). In our series, no SSCPs were detected in immunonegative cancers or matched normal skin samples and there was no difference in the number of SSCPs present in RARs and ICPs. Direct DNA sequencing of one SCC with a SSCP mutation in exon 7 revealed a C–T transition at codon 248 (Figure 3). SSCPs were detected in

**Figure 2** Histological distribution of accumulated p53. **a**, Severely dysplastic keratosis (left) is associated with strong p53 immunostaining as compared with negative normal epidermis (right). **b**, p53 immunostaining is localised to the dysplastic basal cells in actinic keratosis. **c**, Dysplastic basal cells are positive for p53 while the specialised cells in appendages are negative. **d**, Nuclear localisation of p53 in an invasive squamous cell carcinoma from a RAR. p53 immunocytochemistry was performed using PAb Do-7 and a standard ABC horseradish peroxidase technique.

**Table II** Correlation between presence of HPV DNA and accumulated p53 in cutaneous lesions from RARs and ICPs

| | *RARs* | | | | *ICPs* | | | |
|---|---|---|---|---|---|---|---|---|
| | *HPV+* | | *HPV−* | | *HPV+* | | *HPV−* | |
| *Histology* | *p53+*[a] | *p53−* | *p53+*[a] | *p53−* | *p53+*[a] | *p53−* | *p53+*[a] | *p53−* |
| US | 0/3 | 3/3 | 5/16 | 11/16 | 1/1 | 0/1 | 2/11 | 9/11 |
| VWs | 1/10 | 9/10 | 0/5 | 5/5 | 1/5 | 4/5 | 1/1 | 0/1 |
| Ks | 4/10 | 6/10 | 6/19 | 13/19 | 0/3 | 3/3 | 3/11 | 8/11 |
| IECs | 8/12 | 4/12 | 13/20 | 7/20 | 2/4 | 2/4 | 13/24 | 11/24 |
| SCCs | 7/15 | 8/15 | 10/16 | 6/16 | 3/5 | 2/5 | 4/10 | 6/10 |

[a]Sections with staining in >10% of nuclei in the lesion (grade 2 and 3) were scored as positive. RAR, renal allograft recipient; ICP, immunocompetent patient; US, uninvolved, sun-exposed skin; VW, viral wart; K, keratosis; IEC, intraepidermal carcinoma; SCC, squamous cell carcinoma

**Table III** SSCP analysis of immunopositive and immunonegative tumours from RARs and ICPs

| | | *No. of ICC positive and negative lesions exhibiting SSCPs* | | | |
|---|---|---|---|---|---|
| *Patients* | *Histology* | *Neg* | *Grade 1* | *Grade 2* | *Grade 3* |
| RARs | SCC | 0/1 | 0/3 | 0/2 | 3/3 |
| | IEC | 0/3 | 0/1 | 0/1 | 1/2 |
| | Normal | 0/5 | 0/2 | – | – |
| ICPs | SCC | – | 0/2 | 0/2 | 0/2 |
| | IEC | 0/2 | 0/1 | 1/1 | 1/2 |
| | Normal | 0/5 | – | – | – |

ICC, immunocytochemical; SSCPs, p53 mutations as detected by SSCP analysis; grade 1, <10%, grade 2, 10–50%; grade 3, ⩾50% of cells in a lesion showing positive nuclear staining; RAR, renal allograft recipient; ICP, immunocompentent patient, SCC, squamous cell carcinoma; IEC, intraepidermal carcinoma.

three HPV-positive malignancies and one HPV-negative malignancy.

**Figure 3** **a**, Examples of SSCP mutations in exons 5 and 7 of p53 in cutaneous malignancies from RARs and ICPs. Using SSCP analysis, a single base change, such as a point mutation, is visualised as a band of altered migration (as indicated by arrows) in a polyacrylamide gel. Samples 1 and 16 = squamous cell carcinomas from renal allograft recipients, both with grade 3 staining by immunocytochemistry (ICC); sample 36 = an intraepidermal carcinoma from an immunocompetent patient with grade 2 staining by ICC. **b**, Direct DNA sequencing of exon 7 from sample 1 showing a C–T transition at codon 248 of p53.

### Discussion

*p53 accumulation and progression in cutaneous carcinogenesis*

In this study we have demonstrated the presence of accumulated p53 in over 50% of cutaneous SCCs from both RARs and ICPs suggesting that p53 may play a role in skin carcinogenesis in both populations. This detection level is in broad agreement with previously reported results for SCC in ICPs in which it has ranged from 15% to 56% of lesions (Gusterson *et al*., 1991; McGregor *et al*., 1992; Ro *et al*., 1992). A striking feature of our study was the increase in prevalence and extent of staining which occurred as lesions progressed through the histological spectrum of neoplasia. Indeed, there was a close correlation between the extent of staining in these lesions and the severity of dysplasia. These results strongly suggest that in skin carcinogenesis, in both RARs and ICPs, accumulation of p53 represents an important step in malignant progression. This hypothesis is supported by recent studies of skin carcinogenesis in p53 null mice, in which inactivation of p53 specifically associates with progression of benign papillomas to SCCs (Kemp *et al*., 1993). It is important to note, however, that the occurrence of p53 immunoreactivity does not always equate with acquisition of the malignant state since in ICPs accumulated p53 can be demonstrated in solar keratoses, of which only a small proportion progress to invasive carcinoma (Marks *et al*., 1986). Clearly, other genetic events must contribute to the development of invasive skin malignancies. In this context it is also of interest that we found a small number of SCCs showing p53 staining in superficial dysplastic epidermis and adjacent areas of IEC but not in contiguous tongues of invasive SCC. One possible explanation for this may be that gross chromosomal deletions, involving 17p, have occurred in more invasive malignant elements, abolishing all p53 gene expression.

*HPV and p53 in cutaneous lesions from RARs and ICPs*

E6 oncoproteins from HPV types 16 and 18 can bind to and induce rapid degradation of wild-type p53 (Scheffner *et al*., 1990; Werness *et al*., 1990). From observations in anogenital cancers it has been proposed that p53 inactivation occurs either by complexing of wild-type p53 with such viral oncoproteins or, in the absence of virus, by mutational loss of gene function (Crook *et al*., 1991, 1992; Scheffner *et al*., 1991, 1992). This concept, however, remains controversial, and other workers have failed to confirm these suggestions (Busby-Earle *et al*., 1993; Cooper *et al*., 1993). We have recently reported the prevalence of HPV in cutaneous lesions from RARs (Stark *et al*., 1994) and suggested that the mechanism by which HPV contributes to skin carcinogenesis may differ from that proposed for anogenital cancer. The present study confirms our previous findings in that we have failed to demonstrate any relationship between the presence or absence of HPV DNA and accumulated p53 in dysplastic or frankly malignant skin lesions from RARs or ICPs. Moreover, p53 mutations were detected by SSCP analysis in both HPV-positive and -negative malignancies. Recently, it has also been demonstrated that the E6 oncoprotein from skin-associated HPV type 8 does not bind to p53, unlike its HPV 16 or 18 equivalent (Steger & Pfister, 1992). Therefore, if HPV is involved in cutaneous carcinogenesis, it must be presumed to act by a different mechanism from that found in anogenital cancer.

*p53 mutations in cutaneous carcinogenesis*

In this study SSCP analysis was used to demonstrate mutations in the p53 gene. Although the precise sensitivity of this technique is presently unknown, a recent study in our laboratory involving human colorectal cancer, in which both SSCP analysis and direct sequencing were performed, indicates that approximately 80% of p53 mutations can be detected by SSCP analysis (Cripps *et al.*, manuscript in preparation). The detection of p53 mutations in 21% of SCCs/IECs in our series is in agreement with previous reports for cutaneous cancer (Pierceall *et al.*, 1991; Ro *et al.*, 1992; Campbell *et al.*, 1993*a*, *b*). With one exception, these mutations occurred in exons 5 and 7, in keeping with the suggestion that these exons contain mutational hotspots for most human malignancies (Brash *et al.*, 1991; Hollstein *et al.*, 1991; Pierceall *et al.*, 1991; Campbell *et al.*, 1993*a*; Levine, 1993). Molecular analysis of p53 mutations has previously suggested that the pattern of nucleotide alterations may be tissue dependent and related to the type of mutagenic agent involved (Harris, 1991; Vogelstein & Kinzler, 1992). For instance, CC to TT double-base changes are almost exclusively associated with UV-induced DNA damage (Brash *et al.*, 1991). It is of interest, therefore, that the SCC in our series that was sequenced was found to contain a C-T transition at the codon 248 mutational hotspot, implicating UV radiation in its genesis.

### *p53 immunocytochemical detection and gene mutations*

Immunocytochemistry has been proposed as a rapid and simple means of identification of p53 gene mutations. In the majority of tumours, good correlation has been observed between the presence of immunocytochemically stable p53 and gene mutations determined by sequencing or other methods (Gannon *et al.*, 1990; Iggo *et al.*, 1990; Bodner *et al.*, 1992). However, it is also recognised that immunocytochemically detectable levels of wild-type p53 may occur in response to DNA injury, and that some p53 mutations do not result in immunocytochemical demonstration of p53 protein (Bodner *et al.*, 1992; Oliner *et al.*, 1992; Wynford-Thomas, 1992; Hall *et al.*, 1993, Lane, 1993). In this study, SSCP analysis of exons 5-8 detected mutations in 6/15 (40%) immunopositive malignancies, with most mutations occurring in tumours with the largest number of positive cells (grade 3 lesions). This indicates that immunocytochemical detection of p53 does not always signify the presence of p53 gene mutations in skin cancers, particularly where there are relatively few positive cells. While the possibility remains that mutations may have occurred in exons other than 5-8, our own experience and that of others studying other common cancers suggests that this is likely to account for only a small proportion of cases. This implies that additional factors may contribute to the accumulation of p53 during the development of at least some skin cancers. Recently, the product of the *mdm*-2 gene, which is overexpressed in osteosarcomas, has been shown to bind to and inactivate p53 (Momand *et al.*, 1992; Oliner *et al.*, 1992). It is possible that similar proteins may be present in transformed epidermal cells, complexing with wild-type p53 and rendering it detectable by immunocytochemical methods. The identity of such proteins and their role in the accumulation of p53 and subsequent development of skin cancer remain to be established.

We would like to thank the Scottish Home and Health Department for funding this research, Robert Morris and John Lauder for technical advice and Jill Bubb and Andrew Wyllie for useful discussions.

### References

ALLOUB, M.I., BARR, B.B.B., MCLAREN, K.M., SMITH, I.W., BUNNEY, M.H. & SMART, G.E. (1989). Human papillomavirus and lower genital neoplasia in renal transplant patients. *Obstet. Gynecol.*, **68**, 251-258.

ARENDS, M.J., DONALDSON, Y.K., DUVALL, E., WYLLIE, A.H. & BIRD, C.C. (1991). HPV in full thickness cervical biopsies: high prevalence in CIN 2 and CIN 3 detected by a sensitive PCR assay. *J. Pathol.*, **165**, 301-309.

BAKER, S.J., FEARON, E.R., NIGRO, J.M., HAMILTON, S.R., PREISINGER, A.C., JESSUP, J.M., VAN TUINEN, P., LEDBETTER, D.H., BARKER, D.F., NAKAMURA, Y., WHITE, R. & VOGELSTEIN, B. (1989). Chromosome 17p deletions and p53 gene mutations in colorectal carcinomas. *Science*, **249**, 912-915.

BANKS, L., MATLASHEWSKI, G. & CRAWFORD, L. (1986). Isolation of human-p53-specific monoclonal antibodies and their use in the studies of human p53 expression. *Eur. J. Biochem*, **159**, 529-534.

BARR, B.B.B., BENTON, E.C., MCLAREN, K.M., BUNNEY, M.H., SMITH, I.W., BLESSING, K. & HUNTER, J.A.A. (1989). Human papilloma virus infection and skin cancer in renal allograft recipients. *Lancet*, **i**, 124-129.

BENTON, E.C., SHAHIDULLAH, H. & HUNTER, J.A.A. (1992). Human papillomavirus in the immunosuppressed. *Papillomavirus Rep.*, **3**, 23-26.

BLESSING, K., MCLAREN, K.M., BENTON, E.C., BARR, B.B., BUNNEY, M.H., SMITH, I.W. & BEVERIDGE, G.W. (1989). Histopathology of skin lesions in renal allograft in recipients - an assessment of viral features and dysplasia. *Histopathology*, **14**, 129-139.

BLOHME, I. & LARKO, O. (1984). Premalignant and malignant skin lesions in renal transplant patients. *Transplantation*, **37**, 165-167.

BODNER, S.M., MINNA, J.D., JENSEN, S.M., D'AMICO, D., CARBONE, D., MITSUDOMI, T., FEDORKO, J., BUCHHAGEN, D.L., NAU, M.M., GAZDAR, A.F. & LINNOILA, R.I. (1992). Expression of mutant p53 proteins in lung cancer correlates with the class of p53 gene mutation. *Oncogene*, **7**, 743-749.

BOYLE, J., MACKIE, R.M., BRIGGS, J.D., JUNOR, B.J.R. & AITCHISON, T.C. (1984). Cancer, warts and sunshine in renal transplant patients. A case control study. *Lancet*, **i**, 702-705.

BRASH, D.E., RUDOLPH, J.A., SIMON, J.A., LIN, A., MCKENNA, G.J., BADEN, H.P., HALPERIN, A.J. & PONTEN, J. (1991). A role for sunlight in skin cancer: UV-induced p53 mutations in squamous cell carcinoma. *Proc. Natl Acad. Sci. USA*, **88**, 10124-10128.

BURNS, J.E., BAIRD, L.J., CLARK, P.A., BURNS, K., EDINGTON, K., CHAPMAN, C., MITCHELL, C.R., ROBERTSON, G., SOUTAR, D. & PARKINSON, E.K. (1993). Gene mutations and increased levels of p53 protein in human squamous cell carcinomas and their cell lines. *Br. J. Cancer*, **67**, 1274-1284.

BUSBY-EARLE, R.M.C., STEEL, C.M. & BIRD, C.C. (1993). Cervical carcinoma: low frequency of allele loss at loci implicated in other common malignancies. *Br. J. Cancer*, **67**, 71-75.

CAMPBELL, C., QUINN, A.G., RO, Y.-S., ANGUS, B. & REES, J.L. (1993*a*). p53 mutations are a common and early event which precede tumour invasion in squamous cell neoplasia of the skin. *J. Invest. Dermatol.*, **100**, 746-748.

CAMPBELL, C., QUINN, A.G., ANGUS, B. & REES, J.L. (1993*b*). The relation between p53 mutation and p53 immunostaining in non-melanoma skin cancer. *Br. J. Dermatol.*, **129**, 235-241.

CLARKE, A.R., PURDIE, C.A., HARRISON, D.J., MORRIS, R.G., BIRD, C.C., HOOPER, M.L. & WYLLIE, A.H. (1993). Thymocyte apoptosis induced by p53-dependent and independent pathways. *Nature*, **362**, 849-852.

COOPER, K., HERRINGTON, C.S., EVANS, M.F., GATTER, K.C. & MCGEE, J.O.D. (1993). p53 antigen in cervical condylomata, intraepithelial neoplasia and carcinoma: relationship to HPV infection and integration. *J. Pathol.*, **171**, 27-34.

CROOK, T., WREDE, D., TIDY, J.A., SCHOLFIELD, J., CRAWFORD, L. & VOUSDEN, K.H. (1991). Status of c-myc, p53 and retinoblastoma genes in human papillomavirus positive and negative squamous cell carcinomas of the anus. *Oncogene*, **6**, 1251-1257.

CROOK, T., WREDE, D., TIDY, J.A., MASON, W.P., EVANS, D.J. & VOUSDEN, K.H. (1992). Clonal p53 mutation in primary cervical cancer: association with human papillomavirus-negative tumours. *Lancet*, **339**, 1070-1073.

DEBBAS, M. & WHITE, E. (1993). Wild type p53 mediates apoptosis by E1A, which is inhibited by E1B. *Genes Dev.*, **71**, 546-554.

DONEHOWER, L.A., HARVEY, M., SLAGLE, B.L., MCARTHUR, M.J., MONTGOMERY, C.A., BUTEL, J.S. & BRADLEY, A. (1992). Mice deficient for p53 are developmentally normal but susceptible to spontaneous tumours. *Nature*, **356**, 215-221.

GANNON, J.V., GREAVES, R., IGGO, R. & LANE, D.P. (1990). Activating mutations in p53 produce a common conformational effect. A monoclonal antibody specific for the mutant form. *EMBO J.*, **9**, 1595-1602.

GUSTERSON, B.A., ANBAZHAGAN, R., WARREN, W., MIDGELY, C., LANE, D.P., O'HARE, M., STAMPS, A., CARTER, R. & JAYATILAKE, H. (1991). Expression of p53 in premalignant and malignant squamous epithelium. *Oncogene*, **6**, 1785–1789.

HALL, P.A., MCKEE, P.H., DU, P., MANAGE, H., DOVER, R. & LANE, D.P. (1993). High levels of p53 protein in UV-irradiated normal human skin. *Oncogene*, **8**, 203–207.

HARRIS, A.L. (1991). Telling changes of base. *Nature*, **350**, 377–378.

HARTWELL, L. (1992). Defects in a cell cycle checkpoint may be responsible for the genomic instability of cancer cells. *Cell*, **71**, 543–546.

HOLGATE, C.S., JACKSON, P., POLLARD, K., LUNNY, D. & BIRD, C.C. (1986). Effect of fixation on T and B lymphocyte surface membrane antigen demonstration in paraffin processed tissue. *J. Pathol.*, **149**, 293–300.

HOLLSTEIN, M., SIDRANSKY, D., VOGELSTEIN, B. & HARRIS, C.C. (1991). P53 mutations in human cancers. *Science*, **253**, 49–53.

IGGO, R., GATTER, K., BARTEK, J., LANE, D.P. & HARRIS, A.L. (1990). Increased expression of mutant forms of p53 oncogene in primary lung cancer. *Lancet*, **335**, 675–679.

KASTAN, M.B., ONYEKWERE, O., SIDRANSKY, D., VOGELSTEIN, B. & CRAIG, R.W. (1991). Participation of p53 protein in the cellular response to DNA damage. *Cancer Res.*, **51**, 6304–6311.

KEMP, C.J., DONEHOWER, L.A., BRADLEY, A. & BALMAIN, A. (1993). Reduction of p53 gene dosage does not increase initiation or promotion but enhances malignant progression of chemically-induced skin tumours. *Cell*, **74**, 813–822.

LANE, D.P. (1992). p53, guardian of the genome. *Nature*, **358**, 15–16.

LANE, D.P. (1993). A death in the life of p53. *Nature*, **362**, 786–787.

LEVINE, A.J. (1993). The p53 tumour suppressor gene and product, 11th Ernst Klenk Lecture. *Biol. Chem. Hoppe-Seyler*, **374**, 227–233.

MARKS, R., FOLEY, P., GOODMAN, G., HAGE, B.H. & SELWOOD, T.S. (1986). Spontaneous remission of solar keratoses – the case for conservative management. *Br. J. Dermatol.*, **115**, 649–655.

MCGREGOR, J.M., YU, C.C.-W., DUBLIN, E.A., LEVISON, D.A. & MACDONALD, D.M. (1992). Aberrant expression of p53 tumour-suppressor gene in non-melanoma skin cancer. *Br. J. Dermatol.*, **127**, 463–469.

MILNER, J. & MEDCALF, E.A. (1991). Cotranslation of activated mutant p53 with wild type drives the wild type P53 protein into a mutant conformation. *Cell*, **65**, 774–785.

MILNER, J. & COOK, A. (1986). Visualisation, by immunocytochemistry, of p53 at the plasma membrane of both non-transformed and SV40-transformed cells. *EMBO J.*, **9**, 2885–2889.

MOMAND, J., ZAMBETTI, G.P., OLSON, D.C., GEORGES, D.L. & LEVINE, A.J. (1992). The mdm-2 oncogene product forms a complex with the p53 protein and inhibits p53 mediated transactivation. *Cell*, **69**, 1237–1245.

MONTENARH, M. (1992). Biochemical properties of the growth suppressor oncoprotein p53. *Oncogene*, **7**, 1673–1680.

MORAN, E. (1993). Interaction of adenoviral proteins with pRB and p53. *FASEB J.*, **7**, 880–885.

NIGRO, J.M., BAKER, S.J., PREISINGER, A.C., JESSUP, J.M., HOSTETTER, R., CLEARY, K., BIGNER, S.H., DAVIDSON, N., BAYLIN, S., DEVILEE, P., GLOVER, T., COLLINS, F.S., WESTON, A., MODALI, R., HARRIS, C.C. & VOGELSTEIN, B. (1989). Mutations in the p53 gene occur in diverse human tumour types. *Nature*, **342**, 705–708.

OLINER, J.D., KINZLER, K.W., MEITZER, P.S., GEORGES, D.L. & VOGELSTEIN, B. (1992). Amplification of a gene encoding a p53-associated protein in human sarcomas. *Nature*, **358**, 80–83.

PIERCEALL, W.E., MUKHOPADHYAY, T., GOLDBERG, L.H. & ANANTHASWAMY, H.N. (1991). Mutations in the p53 tumour suppressor gene in human cutaneous squamous cell carcinomas. *Mol. Carcinogen.*, **4**, 445–449.

PURDIE, C.A., O'GRADY, J., PIRIS, J., WYLLIE, A.H. & BIRD, C.C. (1991). p53 expression in colorectal tumours. *Am. J. Pathol.*, **138**, 807–813.

PURDIE, C.A., HARRISON, D.J., PETER, A., DOBBIE, L., WHITE, S., HOWIE, S.E.M., SALTER, D.M., BIRD, C.C., WYLLIE, A.H., HOOPER, M.L. & CLARKE, A.R. (1994). Tumour incidence, spectrum and ploidy in mice with a large deletion in the p53 gene. (in press).

RO, Y.-S., VOJTESEK, B., COOPER, P.N., LEE, J.A., HARRISON, D., ANGUS, B., REES, J., HORNE, C.H.W. & LANE, D.P. (1992). p53 protein expression in benign and malignant squamous and melanocytic skin tumours – an immunohistochemical study. *J. Invest. Dermatol.*, **98**, 540–544.

RUDLINGER, R., SMITH, I.W., BUNNEY, M.H. & HUNTER, J.A.A. (1986). Human papillomavirus infections in a group of renal transplant recipients. *Br. J. Dermatol.*, **115**, 681–692.

SAMBROOK, J., FRITSCH, E.F. & MANIATIS, T. (1989). *Molecular Cloning. A Laboratory Manual*, 2nd edn, pp. E3–E4. Cold Spring Harbor Laboratory Press: Cold Spring Harbor, NY.

SCHEFFNER, M., WERNESS, B.A., HULBREGTSE, J.M., LEVINE, A.J. & HOWLEY, P.M. (1990). The E6 oncoprotein encoded by human papillomavirus types 16 and 18 promotes the degradation of p53. *Cell*, **63**, 1129–1136.

SCHEFFNER, M., MUNGER, K., BYRNE, J.C. & HOWLEY, P.M. (1991). The state of the p53 and retinoblastoma genes in human cervical carcinoma cell lines. *Proc. Natl Acad. Sci. USA*, **88**, 5523–5527.

SCHEFFNER, M., TAKAHASHI, T., HUIBREGTSE, J.M., MINNA, J.D. & HOWLEY, P.M. (1992). Interaction of the human papillomavirus type 16 E6 oncoprotein with wild-type and mutant human p53 proteins. *J. Virol*, **66**, 5100–5105.

SHUTTLEWORTH, D., MARKS, R., GRIFFIN, P.J.A. & SALAMAN, J.R. (1987). Dysplastic epidermal change in immunosuppressed patients with renal transplants. *Q.J. Med.*, **243**, 609–616.

SRIVASTAVA, S., ZOU, Z., PIROLLO, K., BLATTNER, W. & CHANG, E.H. (1990). Germ-line transmission of a mutated p53 gene in a cancer-prone family with Li-Fraumeni syndrome. *Nature*, **348**, 747–749.

STARK, L.A., ARENDS, M.J., MCLAREN, K.M., BENTON, E.C., SHAHIDULLAH, H., HUNTER, J.A.A. & BIRD, C.C. (1994). Prevalence of human papillomavirus DNA in cutaneous neoplasms from renal allograft recipients supports a possible viral role in tumour promotion. *Br. J. Cancer*, **69**, 222–229.

STEGER, G. & PFISTER, H. (1992). *in vitro* expressed HPV 8 E6 protein does not bind p53. *Arch. Dermatol.*, **125**, 355–360.

STREILEIN, J.W. (1991). Immunogenetic factors in skin cancer. *New Engl J. Med.*, **325**, 885–886.

UNGER, T., NAU, M.N., SEGAL, S. & MINNA, J.D. (1992). p53: a transdominant regulator of transcription whose function is ablated by mutations occurring in human cancer. *EMBO. J.*, **11**, 1383–1390.

VOGELSTEIN, B. & KINZLER, K.W. (1992). p53 function and dysfunction. *Cell*, **70**, 523–526.

VOJTESEK, B., BARTEK, J., MIDGLEY, C.A. & LANE, D.P. (1992). An immunochemical analysis of the human nuclear phosphoprotein p53 new monoclonal antibodies and epitope mapping using recombinant p53. *J. Immunol. Methods*, **151**, 237–244.

WERNESS, B.A., LEVINE, A.J. & HOWELY, P.M. (1990). Association of human papillomavirus types 16 and 18 E6 proteins with p53. *Science*, **248**, 76–79.

WYNFORD-THOMAS, D. (1992). p53 in tumour pathology: can we trust immunocytochemistry? *J. Pathol.*, **166**, 329–330.

YEW, P.R. & BERK, A.J. (1992). Inhibition of p53 transactivation required for transformation by adenovirus early 1B protein. *Nature*, **357**, 82–85.